# Mechanistic and Kinetic Insights into the Interfacial Polymerization of Fluorine-Containing Polyarylate

**DOI:** 10.3390/polym18010031

**Published:** 2025-12-23

**Authors:** Lingli Li, Tiantian Li, Siyu Chen, Jintang Duan, Cailiang Zhang, Xueping Gu, Lianfang Feng

**Affiliations:** 1 Institute of Zhejiang University-Quzhou, Quzhou 324000, China; 2 State Key Laboratory of Chemical Engineering and Low-Carbon Technology, College of Chemical and Biological Engineering, Zhejiang University, Hangzhou 310027, China

**Keywords:** fluorinated polyarylates, interfacial polymerization, kinetics, process characteristic, reaction mechanism

## Abstract

The interfacial polymerization of fluorine-containing polyarylates (F-PAR) represents an important synthetic route for advanced polymeric materials. This work presents a comprehensive mechanistic investigation through integrated kinetic analysis and macromolecular characterization. The polymerization for both F-PAR and its non-fluorinated analogue (M-PAR) follows a two-stage, second-order kinetic profile, with the F-PAR system exhibiting a lower initial rate constant. Kinetic modeling revealed a dynamic reaction locus, transitioning from the bulk organic phase to an indistinguishable regime. The fluorinated system exhibits distinct stage-dependent behavior: initial retardation due to fluorine-induced “nucleophilicity penalty” on bisphenol monomer followed by a kinetic crossover where the growth rate of F-PAR surpasses M-PAR through enhanced oligomer electrophilicity. The terminal stage reveals fundamental divergence, while flexible M-PAR chains sustain accelerated growth via efficient chain-chain coupling, rigid F-PAR chains reach a molecular weight plateau. The incorporation of fluorine enhances thermal stability and optical transparency due to the low polarizability of C-F bonds. This study provides a complete mechanistic roadmap of fluorine’s dynamic role in polymer architecture control.

## 1. Introduction

Polyarylates (PAR) are a class of high-performance specialty engineering plastics known for their exceptional thermal stability, chemical resistance, and mechanical properties [1,2,3]. Incorporating fluorine into PAR molecular chains provides additional characteristics including low surface energy, low dielectric constants, low water absorption, enhanced weatherability, etc., which broadens applications in flexible electronics, 5G/6G substrates, and protective coatings [4,5,6,7]. Post-polymerization fluorination [8] or end-capping [9] methods typically lead to low fluorine content, which may not suffice the stringent demands of certain applications. Direct polycondensation of fluorine-containing monomers can achieve high fluorine content; however, the strong electron-withdrawing effect of fluorine inhibits monomer nucleophilicities, resulting in relatively low-molecular-weight polymers [10,11].

Interfacial polymerization (IP) is an effective method for preparing high-molecular-weight polymers as the reaction occurs at the liquid-liquid interface with elevated local concentrations and high collision probabilities [12,13,14,15,16]. Commercial PAR, such as U-100 (Unitika, Japan), are produced via IP of bisphenol A (BPA) with isophthaloyl/terephthaloyl chloride (IPC/TPC) [17]. Fluorine-containing polyarylates (F-PAR) were prepared as well using bisphenol AF (BPAF) or other fluorine-containing monomers [4,18,19,20,21]. Despite these advancements, differences remain in the reported reaction mechanism, kinetics, and the effect of fluorine on these reactions.

The prevailing mechanism suggests that bisphenolate ions, facilitated by phase transfer catalysts (e.g., triethylbenzylammonium chloride, TEBAC), diffuse from the aqueous phase into the organic phase to react with acyl chlorides. Different from polyamide synthesis via IP, i.e., nylon 66, where the reaction occurs at the interface as evidenced by instant precipitation of high-molecular-weight polymers upon contact, the exact reaction location for PAR synthesis is ambiguous because the resulting polymers are soluble and the reaction takes place in a slower rate. Brzozowski et al. [22] and Tsai et al. [23,24] have reported a second-order reaction with respect to diacid chloride and bisphenolate concentration, respectively. Diffusion of bisphenolate to the organic phase was suggested as the rate-limiting step. Conversely, Wang et al. [25] concluded the reaction in the organic bulk as the controlling step based on a linear relationship of *C*_BPA_/([−d*C*]_BPA_/d*t*) vs. 1/*C*_BPA_. It is noteworthy that these findings are primarily based on data from the initial 20 min, after which monomers are nearly depleted. Other studies have provided further insights: Berezkin et al. [26] numerically investigated the reaction zone topology, noting a transition to a kinetics-controlled regime at high conversion; Wolinski et al. [27] demonstrated rapid synthesis in a Taylor-Couette reactor, highlighting the role of mixing intensity.

The incorporation of fluorine-containing monomers, such as BPAF, aims to improve thermal [28], radiation resistance [29], and dielectric properties [7]. However, reported outcomes are contradictory and lack a unified mechanistic explanation. Some studies report a suppressive effect of fluorine on polymerization. For instance, Oishi et al. [19] and Kakimoto et al. [30] consistently reported lower inherent viscosities for polymers derived from BPAF compared to those from BPA. Conversely, other studies have reported an apparently opposite trend. Hsio et al. [21] and Wang et al. [4] documented higher inherent viscosities in polymers prepared with BPAF compared to those from fluorine-free bisphenols. These differences remain poorly understood and are often attributed to undefined differences in reaction conditions (e.g., solvent, monomer ratio, stirring rate), making direct comparison and mechanistic extraction difficult.

This work aims to bridge this gap through a comprehensive multi-scale analysis of the IP process of a F-PAR, directly compared with its non-fluorinated analogue (M-PAR). Reaction kinetics, evolutionary molecular weight analysis, and first-principle calculations are systematically integrated to deconvolute the mechanism. Our specific objectives are to: (i) determine the reaction order and rate constants while identifying the primary reaction locus via kinetic modeling; (ii) correlate the distinct kinetic profiles with the real-time evolution of molecular weight and dispersity (PDI) across three distinct growth stages; (iii) elucidate the dualistic electronic role of fluorine via electrostatic potential (ESP) analysis; and (iv) correlate the polymer architectures, resulting from the fluorine-governed pathways, with key material properties including thermal stability and optical transparency. This holistic strategy provides insights into how fluorine dictates the polymerization pathway and final polymer architecture.

## 2. Materials and Methods

### 2.1. Materials

TPC (≥99%), IPC (≥98%), BPAF (≥98%), BPA (≥99%), and TEBAC (98%) were supplied by Shanghai Macklin Biochemical Technology Co., Ltd. (Shanghai, China). Dichloromethane (DCM, ≥99.5%), anhydrous ethanol (≥99.7%), HCl (36.0–38.0%), and chloroform (≥99.0%) were obtained from Sinopharm Chemical Reagent Co., Ltd. (Shanghai, China). Sodium hydroxide (NaOH, ≥96%) was sourced from Xilong Scientific (Shantou, China).

### 2.2. Synthesis of PAR

**Figure 1:**
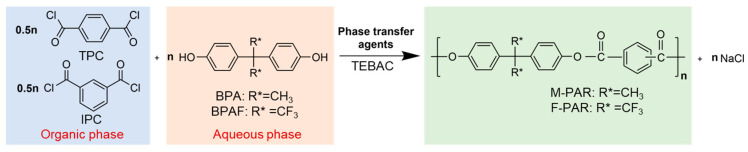
Schematic of polyarylate synthesis through interface polymerization.

The PAR was synthesized via IP. Briefly, 26 mmol of BPAF (or BPA) and 54 mmol of NaOH were charged into a three-necked flask equipped with a mechanical stirrer and an oil bath. The mixture was stirred at 40 °C for 30 min to form a homogeneous disodium bisphenolate aqueous solution. After cooling to 25 °C, 0.15 g TEBAC was added. Simultaneously, an organic solution was prepared by dissolving 12.5 mmol each of IPC and TPC in 60 mL of DCM. The organic solution was then introduced to the aqueous phase to initiate the polymerization at 25 °C. Reaction time was set as 5, 10, 15, 20, 25, 30, 40, 50, 60, 90, and 120 min, respectively. Once the time was reached, the mixture was rapidly separated into aqueous and organic phases. The organic phase was acidified with 10 mL of HCl (1 mol/L) to adjust the pH to 4–5. DCM was removed by azeotropic distillation with 50 mL of hot water at 80 °C. The resulting white solid was washed twice with hot water (80 °C) and anhydrous ethanol, respectively, and dried at 120 °C for 1 h. The reaction scheme is illustrated in Figure 1. In this synthesis, a slight excess of the bisphenol monomer ([diol]/[acyl chloride] = 1.04) was employed to ensure complete consumption of the diacyl chlorides, thereby driving the reaction toward completion and maximizing the polymer molecular weight. An excess of NaOH (54 mmol) was to ensure total deprotonation of the diols (26 mmol). Furthermore, an equimolar ratio of IPC to TPC was utilized, as this is a well-established formulation for producing amorphous PAR.

### 2.3. Characterization

#### 2.3.1. Kinetics Analysis via UV-Vis Spectroscopy

**Figure 2:**
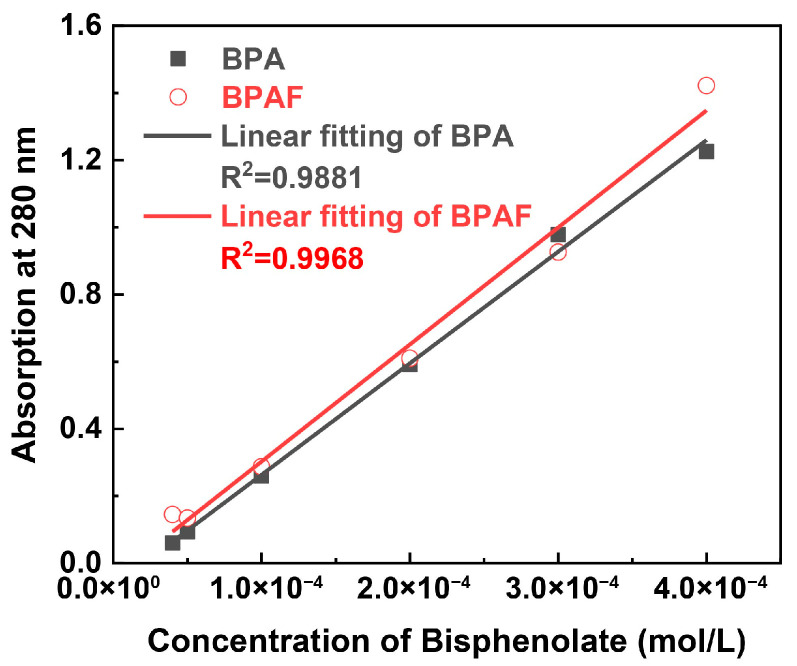
Calibration curve of bisphenolate concentration versus UV absorbance at 280 nm.

The kinetics of monomer consumption were determined by analyzing the aqueous phase of the samples at each time point. The concentration of BPA or BPAF in the aqueous supernatant was quantified using a Shimadzu UV-1800 spectrophotometer. The absorbance at 280 nm was measured, with deionized water as the blank reference. Quantification of BPAF (or BPA) concentration was performed based on a pre-established linear calibration curve (Figure 2) relating absorbance to bisphenolate concentration in aqueous solution.

#### 2.3.2. Gel Permeation Chromatography (GPC) for Molecular Weight and Distribution

The evolution of molecular weight was tracked by analyzing the organic phase of the samples collected over time. The number-average (*M*_n_) and weight-average (*M*_w_) molecular weights of the polymers were measured using an Agilent 1260 Infinity II GPC system (Santa Clara, CA, USA). Chloroform served as the mobile phase, and the measurements were conducted at a sample concentration of 3 mg/mL over a molecular weight range of 0.5 × 10^3^ to 10^7^ g/mol. The system was calibrated with polystyrene standards. PDI was calculated as the ratio of *M*_w_ to *M*_n_.

#### 2.3.3. In-Situ Viscosity Monitoring

The evolution of the organic phase viscosity during polymerization was monitored in situ using a Hydramotion XL7-HT online viscometer (Hydramotion Ltd., Malton, UK). At designated time intervals, the mechanical stirring was paused briefly. The viscometer probe was inserted into the middle layer of the organic phase and allowed to stabilize for 2 min before recording the viscosity value. The probe was then removed, and stirring was resumed to ensure minimal disturbance to the reaction.

#### 2.3.4. ESP Analysis

Molecular simulations for ESP analysis were conducted using the Gaussian 09 program (Linux version, Gaussian, Inc., Wallingford, CT, USA). The calculations employed the B3LYP-D3 functional in conjunction with the 6-31G** basis set. Initial molecular structures were built and optimized using GaussView 5.0. The resulting ESP surfaces were visualized and analyzed to assess the local reactivity and charge distribution.

#### 2.3.5. Thermogravimetric Analysis (TGA)

TGA was performed on a TA Instruments TGA55 thermogravimetric analyzer (TA Instruments, New Castle, DE, USA) to evaluate the thermal stability of the polymers. Measurements were conducted under a nitrogen atmosphere from 50 °C to 700 °C at a constant heating rate of 20 °C/min.

#### 2.3.6. Mechanical Tensile Testing

The tensile properties of the polymer films were determined using a SUNS universal material testing machine (SUNS Industrial Group Co., Ltd., Shenzhen, China)). Dumbbell-shaped specimens (28 mm × 5 mm × 0.15 mm) were cut from solution-cast films and tested at 25 °C with a crosshead speed of 5 mm/min.

#### 2.3.7. Transmittance Test

The optical transparency of the solution-cast films was characterized by UV-Vis spectroscopy. Transmittance spectra were recorded in the 200–700 nm wavelength range using a Shimadzu UV-1800 spectrophotometer (Shimadzu Corporation, Kyoto, Japan), with deionized water as the reference. Colorless and transparent films with a controlled thickness of 80–100 μm, prepared via solution casting, were used for the measurements. The transmittance (*T*) was calculated from the absorbance (*A*) using the formula *T* = 10^−*A*^.

## 3. Results and Discussion

### 3.1. Kinetic Analysis and Mechanistic Elucidation

#### 3.1.1. Determination of Reaction Order and Rate Constants

**Figure 3:**
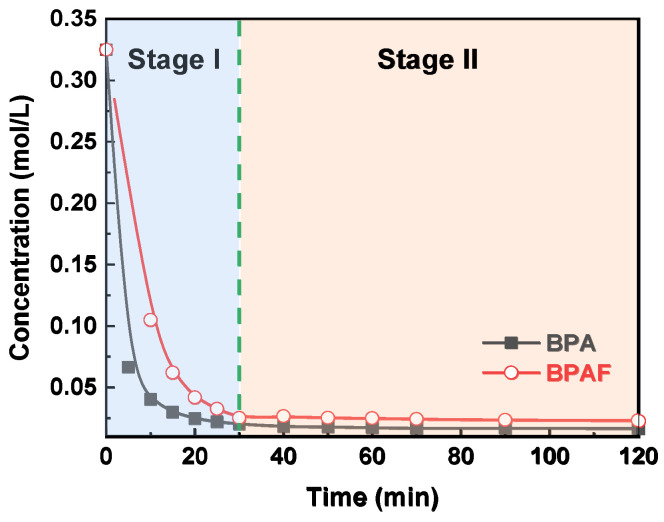
Evolution of aqueous-phase bisphenol concentration as a function of reaction time.

**Figure 4:**
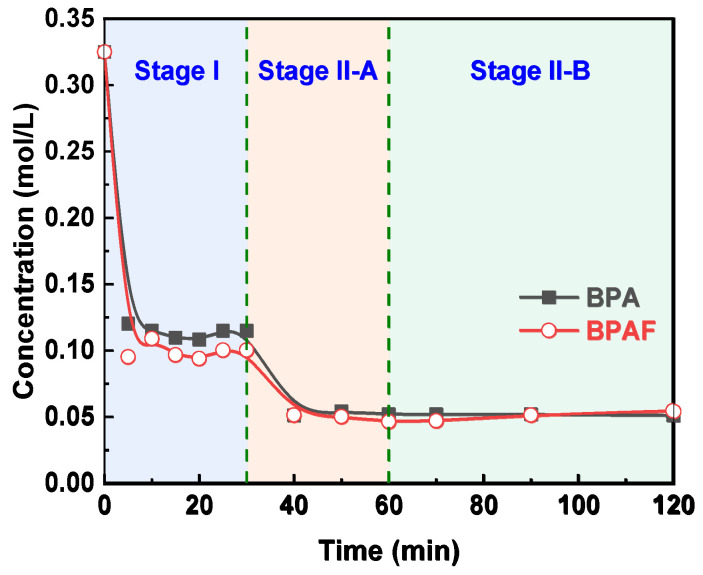
Evolution of bisphenol concentration in the aqueous phase over time in the absence of IPC and TPC.

The reaction kinetics of the IP for both BPA and BPAF were elucidated by monitoring the temporal evolutions of monomer concentration. As illustrated in Figure 3, the concentrations of both monomers decreased rapidly during the initial stage I (0–30 min) before stabilizing in the subsequent stage II (30–120 min). This two-stage behavior is consistent with the mechanism reported by Tsai et al. [23,24], involving a transition from being diffusion-controlled to reaction-controlled. The stabilization of the aqueous-phase monomer concentration during stage II indicates that the system reaches a steady state characterized by a dynamic equilibrium between diffusion and reaction, rather than complete monomer depletion. This conclusion is supported by pure diffusion experiments (Figure 4), wherein BPA/BPAF concentrations stabilized at non-zero values at 10 and 40 min in the absence of IPC/TPC, demonstrating an inherent physical partitioning equilibrium. In the actual interfacial polymerization, the rapid monomer consumption at the reaction locus in stage I creates a large concentration gradient, pulling monomer from the bulk and causing the observed sharp concentration decline. As the reaction transitions to being reaction-controlled in stage II, the monomer consumption rate decreases. Consequently, the rate of monomer supply via diffusion from the bulk adjusts and eventually equals the diminished consumption rate. Thus, while monomers are indeed continuously diffusing from the bulk to the interface and being consumed, the bulk aqueous phase exhibits a macroscopically constant average concentration over the observation period (30–120 min) due to its much larger volume relative to the interfacial region and the now slow consumption rate.

**Figure 5:**
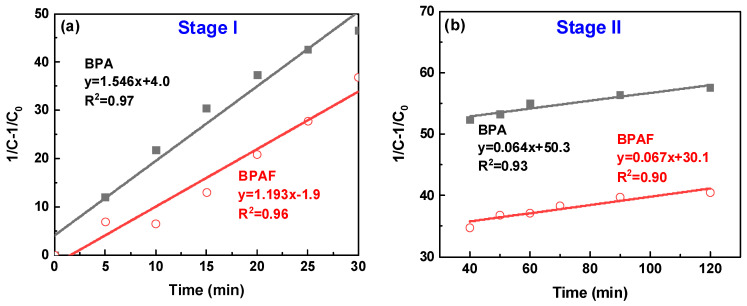
Second-order kinetic plots of (1/C–1/C_0_) versus reaction time for the IP: (**a**) stage I and (**b**) stage II.

**Figure 6:**
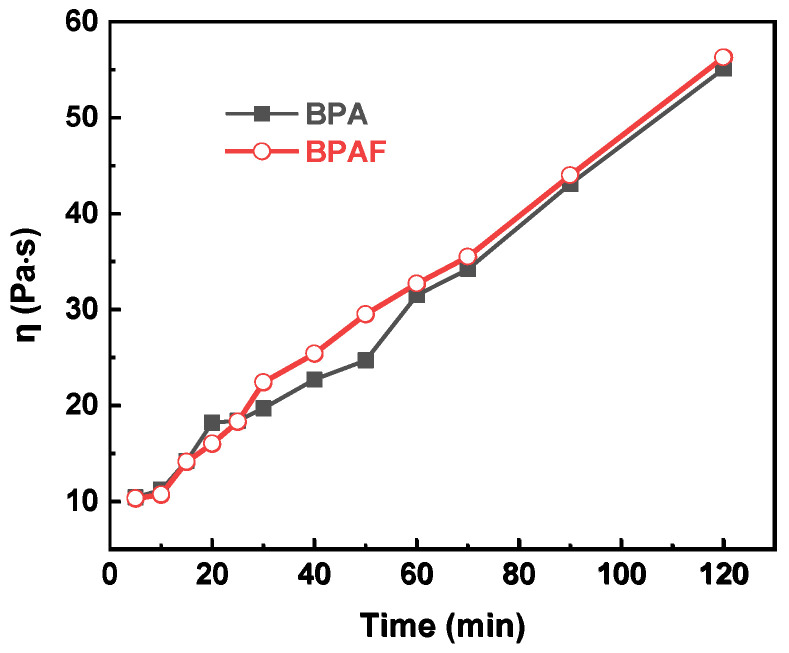
Evolution of organic phase viscosity as a function of reaction time.

To quantitatively deconstruct this behavior, the data were modeled using second-order kinetics. The linear plots of 1/*C*–l/*C*_0_ versus time for the distinct intervals of 0–30 min and 30–120 min (Figure 5) confirm this kinetic model at both stages. The calculated reaction rate constants reveal the dynamic nature of the process: the initial rate constants (1.546 L·mol^−1^·min^−1^ for BPA and 1.193 L·mol^−1^·min^−1^ for BPAF) are approximately an order of magnitude larger than those in the subsequent stage (0.067 and 0.064 L·mol^−1^·min^−1^, respectively). This attenuation in rate is attributed to a transition from a diffusion-controlled regime to a reaction-controlled regime. In the initial phase, the reaction rate is governed by the rapid diffusion of bisphenolate monomers to the interface. As polymerization proceeds, the increased viscosity of the organic phase (Figure 6), resulting from the growing polymer chains, creates a physical barrier that hampers monomer transport, thereby rendering the intrinsic chemical step, the nucleophilic aromatic substitution, as the new rate-determining step.

A pivotal finding from this kinetic analysis is the stage-dependent influence of fluorine. In Stage I, the rate constant for BPAF (1.193 L·mol^−1^·min^−1^) was much lower than that for BPA (1.546 L·mol^−1^·min^−1^). This result provides direct kinetic evidence that the strong electron-withdrawing effect of fluorine imposes a substantial “nucleophilicity penalty” on the monomer, thereby moderating the initial reaction rate, a mechanism further elucidated in Section 3.3. In contrast, the rate constants for both systems converged to nearly identical values in Stage II. This convergence indicates that as the reaction transitions to a reaction-controlled regime, the intrinsic chemical step of the nucleophilic attack becomes the dominant factor for both monomers, thereby diminishing the discriminatory electronic effect of fluorine that was predominant in the initial diffusion-controlled stage.

#### 3.1.2. Identification of the Primary Reaction Locus via Kinetic

**Figure 7:**
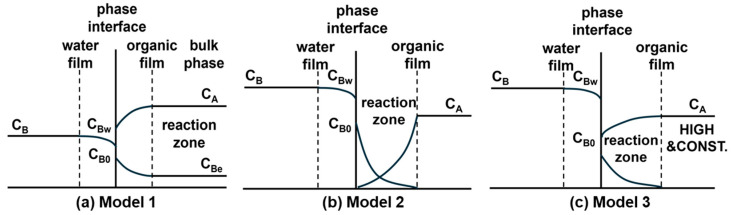
Schematic illustrations of the kinetic models for distinguishing the primary reaction locus in interfacial polycondensation: (**a**) Model 1, (**b**) Model 2, and (**c**) Model 3.

**Figure 8:**
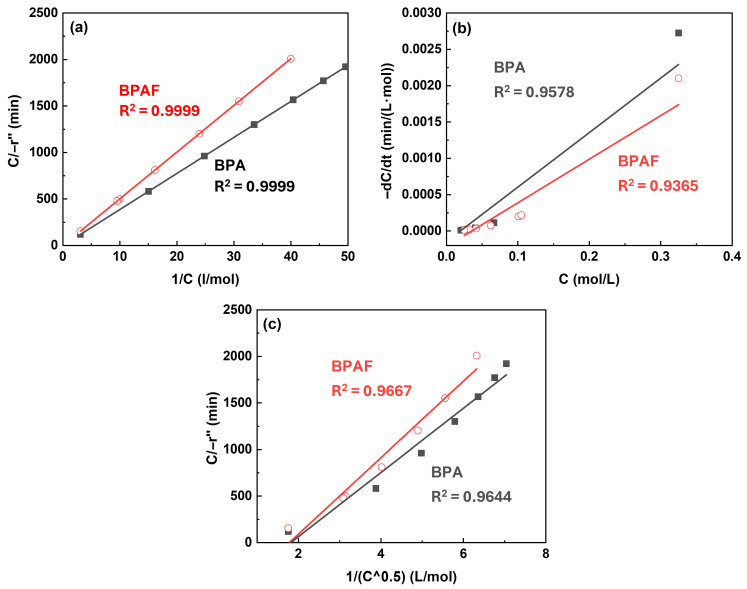
Kinetic model validation plots at Stage I: (**a**) *C*/−*r*″ versus 1/*C* for model 1, (**b**) −d*C*/d*t* versus *C* for model 2, and (**c**) *C*/−*r*″ versus 1/*C*^0.5^ for model 3.

Modeling An important step in elucidating the polymerization mechanism was to identify the dominant reaction zone. To this end, three different kinetic models proposed by Wang et al. [4] was employed, which localize the primary reaction to different zones (Figure 7): the bulk organic phase near the interface (Model 1) or directly at the interface under differing concentration constraints (Models 2 and 3). Following the derivation of corresponding rate equations (Equations (1)–(3), respectively) based on mass balance and chemical kinetics, these models were quantitatively evaluated by the linearity of the plot. During the initial reaction stage (0–30 min), Model 1 exhibited best linear fitting performance (R^2^ > 0.99) compared to Model 2 and Model 3, confirming that the reaction primarily occurs within the bulk organic phase during this period (Figure 8). This finding establishes the initial reaction sequence: diffusion of the aqueous-phase monomer across the interface into the bulk organic phase, followed by nucleophilic substitution within that phase.

(1) $$\frac{C_{BPA\left(F\right)}}{{-r}^{\prime \prime }}=a+\frac{b}{C_{BPA\left(F\right)}}$$

(2) $$-\frac{dC_{BPA\left(F\right)}}{dt}=c\times C_{BPA\left(F\right)}$$

(3) $$-\frac{dC_{BPA\left(F\right)}}{dt}=\frac{d}{e+\frac{f}{\sqrt{C_{BPA\left(F\right)}}}C_{BPA\left(F\right)}}$$

**Figure 9:**
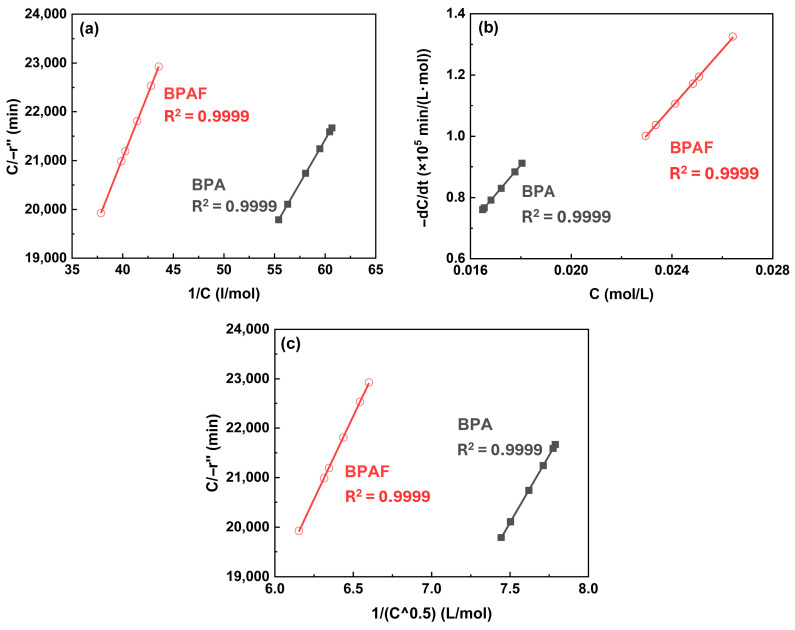
Kinetic model validation plots at Stage II: (**a**) *C*/−*r*″ versus 1/*C* for model 1, (**b**) −d*C*/d*t* versus *C* for model 2, and (**c**) *C*/−*r*″ versus 1/*C*^0.5^ for model 3.

However, in the subsequent stage (30–120 min), the distinction between the models diminished, with all three exhibiting comparable fitting quality (Figure 9). This convergence can be primarily attributed to the depletion of the aqueous-phase bisphenolate monomer, as observed in Figure 3. As the monomer concentration drops to a very low level, the thermodynamic driving force for its phase transfer and the resulting reaction rate diminish substantially. Under such conditions, the inherent assumptions of the different models, which are predicated on distinct concentration gradients and reaction locus, become less discriminating. Consequently, the behavior of system is dominated by the limited availability of the reactant, making it difficult to kinetically distinguish between a reaction occurring predominantly in the bulk organic phase versus one at the interface.

This time-resolved analysis reveals the dynamic nature of IP, demonstrating that the reaction locus evolves from clear bulk organic phase dominance to an indistinguishable regime as monomer conversion advances.

### 3.2. Evolution of Molecular Weight and Distribution: Correlating Kinetics with Polymer Growth

**Figure 10:**
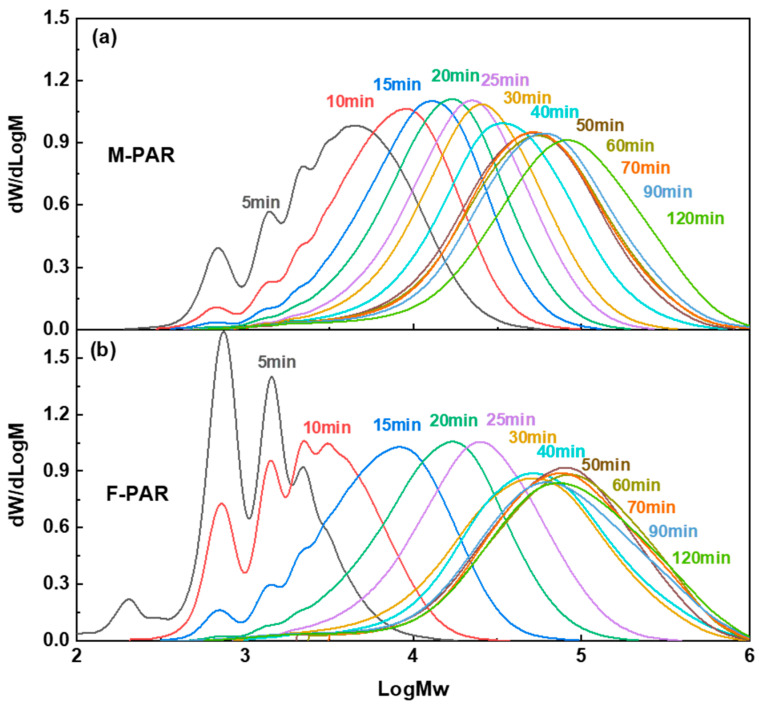
Evolution of GPC profiles with reaction time for (**a**) M-PAR and (**b**) F-PAR.

**Figure 11:**
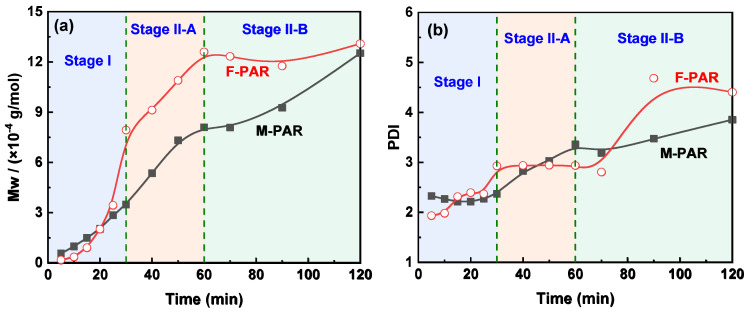
Evolution of (**a**) *M*_w_ and (**b**) PDI with reaction time for M-PAR and F-PAR.

GPC analysis delineates an evolution in molecular weight and dispersity for F-PAR and M-PAR (Figure 10), a progression that directly mirrors their kinetic profiles as shown in Figure 11. During Stage I (0–30 min), the molecular weight growth of F-PAR was significantly slower than that of M-PAR. This observation is directly correlated with, and explained by, the substantially lower reaction rate constant of the BPAF monomer (1.193 L·mol^−1^·min^−1^) compared to the BPA monomer (1.546 L·mol^−1^·min^−1^) in this initial, diffusion-controlled period, consistent with the “nucleophilicity penalty”.

A pivotal shift occurred in Stage II-A (30–60 min). Despite the convergence of the monomeric rate constants for BPAF and BPA to nearly identical values (0.067 vs. 0.064 L·mol^−1^·min^−1^), the molecular weight of F-PAR increased rapidly, with its growth rate far surpassing that of M-PAR. This clear divergence between monomer-level kinetics and macroscopic polymer growth underscores a change in the governing mechanism. The reversal implies that the reaction is no longer dominated by the inherent reactivity of the initial monomers but is instead driven by the enhanced reactivity of the fluorinated oligomeric intermediates, which will be quantitatively explored in Section 3.3.

The terminal stage (Stage II-B, 60–120 min) revealed a difference in polymerization behavior. While F-PAR reached a molecular weight plateau accompanied by dispersity broadening, M-PAR exhibited not only sustained growth but an accelerated rate of molecular weight increase compared to Stage II-A, with only a moderate rise in DPI. This acceleration in M-PAR’s growth is noteworthy, given that the monomer-level rate constants for BPA and BPAF in this stage are nearly identical (0.064 vs. 0.067 L·mol^−1^·min^−1^), indicating that the disparity originates at the macromolecular level.

### 3.3. The Stage-Dependent Role of Fluorine in Polymerization Kinetics and Mechanism

The influence of fluorine on the kinetics and molecular weight development can be traced to its electronic effects. To move beyond phenomenological observation and establish definitive causality, ESP analysis was employed to probe the electronic structures of the monomers and oligomers at each key stage of the reaction.

#### 3.3.1. Monomer Reactivity: The Origin of the “Nucleophilicity Penalty”

**Figure 12:**
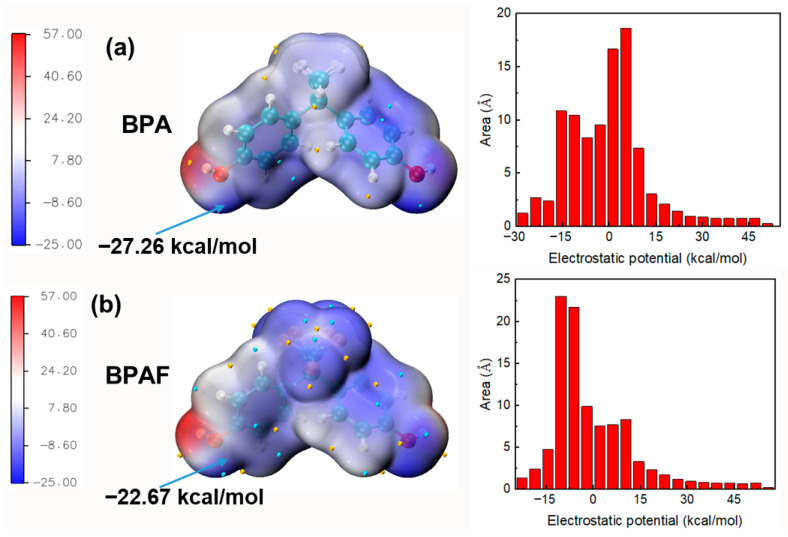
ESP mapped onto the van der Waals surface of (**a**) BPA and (**b**) BPAF. The red and blue regions correspond to more positive and negative ESP values, respectively. The global minima and maxima are indicated by cyan and orange spheres.

The significantly higher reaction rate constant of BPA compared to BPAF in Stage I, resulting in faster molecular weight increase for M-PAR, is primarily governed by the intrinsic reactivity of the monomers rather than their diffusion kinetics. To isolate the role of diffusion, a control experiment monitoring the concentration profiles of BPA and BPAF in the absence of the organic-phase reactant (IPC/TPC) was performed. The results (Figure 4) demonstrated nearly identical, and even slightly faster diffusion for BPAF, effectively eliminating differential aqueous-phase diffusion as a contributing factor. The cause of the kinetic difference is therefore attributed to the electronic structure of the monomers. ESP mapping provides direct quantum-chemical evidence for this conclusion (Figure 12). The ESP value at the hydroxyl oxygen, a key descriptor of nucleophilic strength, is less negative for BPAF (−27.26 kcal/mol) than for BPA (−22.67 kcal/mol). This quantifiable 20.2% reduction in electron density identifies the “nucleophilicity penalty” imposed by the electron-withdrawing fluorine substituents on bisphenol monomers. This penalty accounts for slower nucleophilic attack rate of BPAF, which governs the initial polymerization kinetics and the slower molecular weight buildup of F-PAR.

#### 3.3.2. Oligomer Electrophilicity: The Driver for Accelerated Propagation

The acceleration in molecular weight growth of F-PAR during Stage II-A (30–60 min), which far surpasses that of M-PAR despite nearly identical monomer reaction rate constants, indicates a shift in the reaction mechanism. Two key observations substantiate this conclusion. First, pure diffusion experiments reveal comparable mass transfer kinetics for both bisphenols, as evidenced by the characteristic concentration profile of BPA. The apparent steady state reached at 10 min represents a local interfacial equilibrium. The subsequent concentration decline (30–40 min) results from the slow diffusion of BPA from the interfacial region into the bulk organic phase, which disrupts this local equilibrium and drives further transfer from the aqueous phase until global thermodynamic equilibrium is established. This identical, efficient mass-transfer behavior for both monomers eliminates differential transport as the origin of the different polymer growth. Second, the convergence of monomer consumption rate constants suggests that the reaction is no longer governed by the inherent reactivity of the initial monomers. Instead, the faster growth of F-PAR can be driven by the enhanced reactivity of its fluorinated oligomeric intermediates.

**Figure 13:**
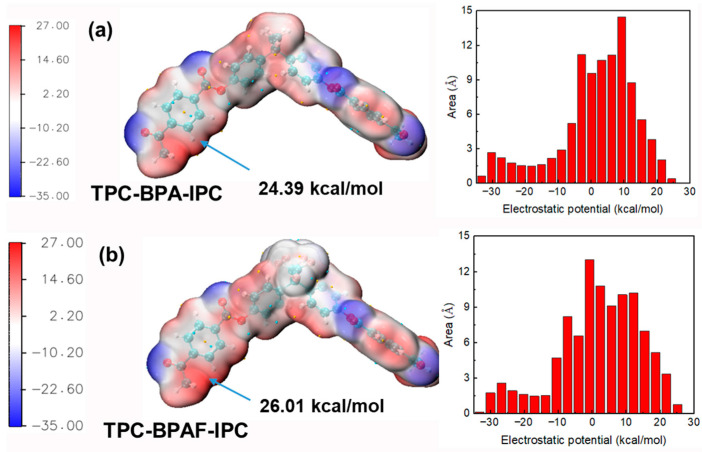
ESP mapped onto the van der Waals surface of (**a**) TPC-BPA-IPC and (**b**) TPC-BPAF-IPC. The red and blue regions correspond to more positive and negative ESP values, respectively. The global minima and maxima are indicated by cyan and orange spheres.

This mechanistic transition is quantitatively explained by ESP analysis of the oligomers (Figure 13). While fluorine withdraws electron density from the monomeric BPAF phenolate, creating a “nucleophilicity penalty”, it exerts an opposite, activating effect on the growing chains. The fluorinated oligomer (TPC-BPAF-IPC) exhibits a higher localized ESP maximum of +26.01 kcal/mol near its benzene ring compared to the +24.39 kcal/mol observed for its non-fluorinated counterpart (TPC-BPA-IPC). This enhanced positive potential, while not at the direct reaction site, exerts a strong electron-withdrawing inductive effect through the conjugated system. This effect increases the partial positive charge, and thus the electrophilicity, on the adjacent carbonyl carbon, making it more susceptible to nucleophilic attack.

As the polymerization progresses, the concentration of these highly electrophilic fluorinated oligomers increases. Their enhanced reactivity enables them to be attacked more efficiently even by the less nucleophilic BPAF phenolate, thereby accelerating chain propagation in Stage II-A. This oligomer-activated mechanism, where the role of fluorine transitions from monomer deactivator to oligomer activator, and explains the rapid increase in molecular weight of F-PAR.

#### 3.3.3. Macromolecular Rigidity: The Determinant of Ultimate Molecular Weight and Dispersity

Stage II-B was marked by a contrast in macromolecular evolution. F-PAR reached a molecular weight plateau accompanied by an increase in dispersity, whereas M-PAR demonstrated sustained and even accelerated growth with only a modest rise in dispersity. This divergence, occurring under conditions of limited monomer diffusion (Figure 3) and nearly identical monomeric rate constants, underscores the governing mechanism stem from chain-chain interaction dynamics.

For M-PAR, its chain flexibility permits a highly efficient chain-chain coupling process. The high local concentration of reactive polymer ends drives this auto-accelerated growth, resulting in the observed rapid molecular weight increase. Conversely, the chains of F-PAR are more rigid, creating a diffusion-limited, kinetically “frozen” state. The molecular origin of this divergence can be traced to the conformational effects induced by fluorine. As demonstrated in polyimide systems [31], the incorporation of a hexafluoroisopropylidene unit leads to a characteristic ratio of 0.787, which is 38.6% lower than the value of 1.281 for a methylene link. This quantifies that fluorinated chains are more extended and conformationally restricted. This reduction in conformational freedom, coupled with a 28.0% larger van der Waals volume, creates a distinctive polymer physics scenario: although the fractional free volume increases significantly by 76.9%, the chains themselves become too rigid to efficiently utilize this space for the segmental motion required for chain-end encounters. This exact mechanism operates in F-PAR, where -CF3 groups similarly restrict chain dynamics, preventing the coupling reactions essential for continued growth. Molecular weight growth is thus arrested.

### 3.4. Influence of Fluorine Incorporation on the Properties of PAR

**Figure 14:**
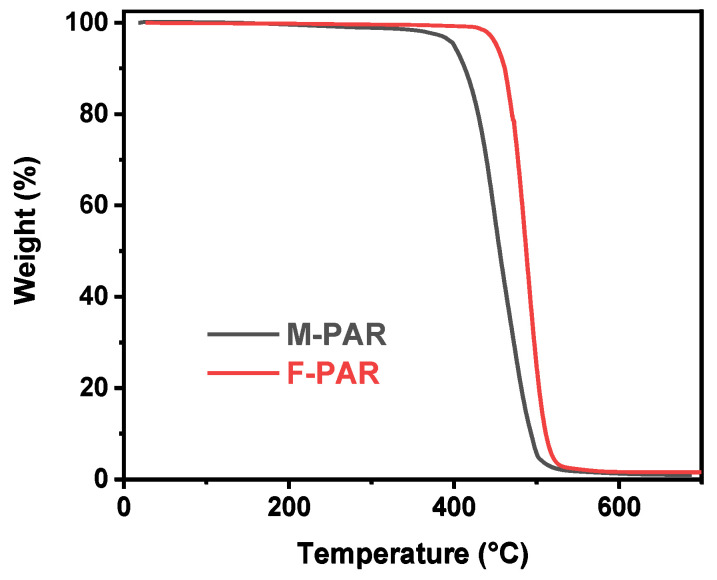
Thermal stability comparison of M-PAR and F-PAR by TGA.

The introduction of fluorine substituents profoundly influences the properties of PAR, encompassing thermal stability, mechanical strength, and optical clarity. The TGA curves in Figure 14 demonstrate the enhanced thermal stability conferred by fluorine. The F-PAR exhibits a 5% mass loss temperature (*T*_d,5%_) of 452 °C, which is markedly higher than the 400 °C recorded for M-PAR. This increase in *T*_d,5%_ is attributed to the high bond dissociation energy of the C-F bond and the increased molecular rigidity, which collectively improve the thermal resistance of the polymer backbone.

The mechanical and optical properties of M-PAR and F-PAR films, summarized in Table 1, reveal a trade-off inherent to fluorine modification. The tensile strength of F-PAR film was measured at 59 MPa, lower than the 74 MPa of the M-PAR film. This reduction in mechanical strength is a direct consequence of the enhanced chain rigidity and the potentially reduced chain packing efficiency induced by the bulky-CF3 side groups, which may limit efficient stress transfer between polymer chains. Conversely, the optical properties are improved. The transmittance of the F-PAR film at both 400 nm (*T*_400_) and 450 nm (*T*_450_) is higher than that of the M-PAR film. Furthermore, a blue shift in the cut-off wavelength (*λ*_cut-off_) is observed for F-PAR. These enhanced optical characteristics are likely due to the low polarizability of the C-F bond and the suppression of intermolecular charge-transfer interactions, which collectively reduce light scattering and absorption in the visible region.

To elucidate the structure-property relationships, it is instructive to compare our results with the work of Wang et al. [4] on bisphenol B (BPB)-based PAR with a TPC/IPC ratio of 7:3. A focused comparison on fluorination reveals its architecture-dependent impact. In their BPB/BPAF-based system, replacing the -CH_3_ with -CF_3_ leads to a moderate 3.5% rise in *T*_d,5%_ (from 474 to 491 °C) with a decrease in tensile strength from 58 to 53 MPa. A similar trend is observed in our BPA/BPAF-based system (TPC/IPC = 5:5), where fluorination achieves a substantial 13% enhancement in *T*_d,5%_ (from 400 to 452 °C) while also reducing the tensile strength (from 74 to 59 MPa). This parallel, enhanced thermal stability but moderated mechanical strength, confirms a consistent trade-off associated with the -CF_3_ group across different PAR architectures.

In summary, fluorine incorporation endows PAR with a superior combination of high thermal stability and optical transparency, albeit at the cost of a moderate reduction in mechanical strength. This structure-property relationship provides guidance for tailoring PAR towards specific applications, such as high-temperature or optically clear coatings and films. Furthermore, the incorporation of fluorine is expected to reduce the surface friction coefficient [32], which would broaden the utility of these polymers in tribological applications.

## 4. Conclusions

In summary, this work systematically elucidates the mechanism and kinetics governing the IP of fluorine-containing PAR. The process is characterized by a two-stage, second-order kinetic profile, comprising an initial diffusion-controlled regime followed by a reaction-controlled stage, with the F-PAR system exhibiting a lower rate constant in the initial phase. Kinetic modeling revealed a dynamic reaction locus, evolving from clear bulk organic phase dominance to an indistinguishable regime as monomer conversion advanced.

This kinetic framework directly correlates with a three-stage molecular weight evolution, decoded through mechanistic analysis. In Stage I, the higher reaction rate constant of BPA compared to BPAF resulted in faster molecular weight increase for M-PAR, a direct “nucleophilicity penalty” imposed on the BPAF monomer by the electron-withdrawing fluorine substituents. In Stage II-A (30–60 min), an acceleration in F-PAR’s molecular weight growth occurred despite nearly identical monomer rate constants. This kinetic crossover is driven by the enhanced electrophilicity of the fluorinated oligomeric intermediates, rendering them more susceptible to nucleophilic attack. The most divergence emerged in Stage II-B: F-PAR reached a molecular weight plateau accompanied by dispersity broadening, whereas M-PAR exhibited sustained and even accelerated growth with a controlled dispersity increase. This is attributed to M-PAR’s chain flexibility and the high local concentration of reactive chain ends, which facilitated efficient chain-chain coupling. In contrast, the rigidity of F-PAR chains suppresses this coupling pathway, leading to a stagnant regime in molecular weight.

Consequently, these fluorine-governed polymerization pathways dictate the final material properties. The incorporation of fluorine enhances the thermal stability of PAR, as evidenced by a 52 °C increase in the *T*_d,5%_. It also bestows superior optical transparency, attributed to the low polarizability of C-F bonds. However, these benefits are concurrently accompanied by a reduction in tensile strength, a direct consequence of the chain rigidity imposed by the bulky-CF3 groups.

This study provides a holistic understanding of how fluorine, through its stage-dependent electronic and steric effects, dictates the polymerization pathway from monomer reactivity to ultimate polymer architecture, offering insights for the rational design of high-performance fluorinated polymers.

## Figures and Tables

**Table 1:** Mechanical and optical properties of M-PAR and F-PAR films.

Polymer	Tensile Strength/MPa	*T*_400_/%	*T*_450_/%	*T*_cut-off_/nm
M-PAR	74	87.1	91.2	332
F-PAR	59	91.2	95.5	328

## Data Availability

The original contributions presented in this study are included in the article. Further inquiries can be directed to the corresponding author.
